# Systemic Impact of *Helicobacter pylori*: A Cross‐Sectional Study

**DOI:** 10.1002/jgh3.70169

**Published:** 2025-10-21

**Authors:** Amanda Ferreira Paes Landim Ramos, Silvana Barbosa Santiago, Felipe Augusto de Sousa Moraes, Giovana Alice Sampaio Soares, Gisele Aparecida Fernandes, Maria Paula Curado, Janaina Naiara Germano, Mônica Santiago Barbosa

**Affiliations:** ^1^ Helicobacter pylori Study Center, Department of Biosciences and Biotechnology Institute of Tropical Pathology and Public Health, Federal University of Goiás Goiânia Goiás Brazil; ^2^ Faculty of Medicine, Postgraduate Program in Health Sciences Federal University of Goiás Goiânia Goiás Brazil; ^3^ A.C. Camargo Cancer Center São Paulo São Paulo Brazil

**Keywords:** bacterium, comorbidity, disease, epidemiology

## Abstract

**Background and Aim:**

*Helicobacter pylori* is an oncobacteria that infects about half of the world's population and has a well‐established role in the etiology of gastric diseases. Lately, this infection has also been associated with extragastric diseases, such as neuropsychiatric, cardiovascular, metabolic, hematological, and dermatological comorbidities. Elucidating risk factors for comorbidities can contribute to reducing mortality and public health costs. Therefore, the aim of this study is to investigate the association between *H. pylori* infection and extragastric comorbidities.

**Materials and Methods:**

This is a cross‐sectional hospital‐based case–control study that was conducted in Goiás from 2019 to 2022. The study patients were classified into *H. pylori* ‐negative and *H. pylori* ‐positive groups.

**Results:**

A total of 156 participants were included in the study, and the prevalence of the bacteria was 45.5%. In the *H. pylori* ‐positive group, the most frequent diseases were hypertension, anemia, rheumatic disease, and diabetes. The presence of comorbidities was similar between the groups, with the exception of psychiatric illnesses. The male patients were more likely to be infected with *H. pylori* (odds ratios [ORs] = 2.63, 95% CI: 1.26–5.50), while the *H. pylori* ‐positive group was less likely to have psychiatric illnesses (OR = 0.32, 95% CI: 0.11–0.92).

**Conclusion:**

The prevalence of *H. pylori* infection was 45.5%, and males were more likely to be infected by the bacteria. The most frequent comorbidities in the *H. pylori* ‐positive group were hypertension, anemia, rheumatic disease, and diabetes. *H. pylori* ‐negative patients were more likely to have psychiatric illnesses.

## 1 Introduction

*Helicobacter pylori* ( *H. pylori* ) is a gram‐negative, non‐acidophilic, microaerophilic, pleomorphic, flagellated bacterium [1]. It has a cosmopolitan distribution and colonizes more than half of the world's population. The prevalence of *H. pylori* infection is associated with factors such as ethnicity, lifestyle habits of the population, age, and socioeconomic status [2]. In developing countries such as Brazil, its prevalence rates are higher, reaching levels above 70% in some regions [3, 4].

*H. pylori* infection remains asymptomatic in most cases throughout the life of the host. The outcome of infection depends on the host factors, environmental factors, and bacterial virulence factors. The imbalance of the parasite–host relationship results in the development of diseases, such as gastritis, peptic ulcer, and gastric cancer [5]. In 1994, due to its well‐established role in gastric carcinogenesis, *H. pylori* was classified as a type 1 carcinogen by the International Agency for Research on Cancer (IARC) [6].

In addition to the well‐established role of *H. pylori* infection in gastric diseases, since the 1990s, some studies have shown its association with different extragastric manifestations. Chronic inflammation and changes in gastrointestinal microbiota caused by infection are the main factors that may contribute to the development of chronic noncommunicable diseases (NCDs), among them being neurological, cardiovascular, metabolic, hematological, dermatological, ocular, and allergic diseases [7, 8].

NCDs are a major public health problem worldwide, especially in Brazil. According to the Pan American Health Organization (PAHO), NCDs represent the main cause of death and disability in Latin America, with greater evidence for cerebrovascular diseases, cardiovascular diseases, obesity, and diabetes [9]. According to the World Health Organization (WHO), 73.6% of deaths worldwide in 2019 were attributed to NCDs. In the same year in Brazil, NCDs accounted for 41.8% of all premature deaths among patients between 30 and 69 years of age. Some factors may contribute to the development of these diseases, such as a sedentary lifestyle, alcohol and tobacco consumption, inadequate diet, and *H. pylori* infection, which has recently been proposed [10].

Since data on *H. pylori* infection associated with the presence of comorbidities are limited, the investigation of this relationship and the probable mechanisms are extremely relevant. Therefore, the aim of this study is to investigate the likely association between *H. pylori* infection and extragastric comorbidities in dyspeptic patients of a healthcare institution in Central Brazil.

## 2 Methodology

### 2.1 Study Population

This is a cross‐sectional hospital‐based case–control study that was conducted in the city of Goiânia (Goiás) from 2019 to 2022. Data were collected from patients at the Hospital das Clínicas da Universidade Federal de Goiás (HC/UFG). The inclusion criteria were as follows: patients aged between 18 and 75 years of both genders with gastric complaints and undergoing upper digestive endoscopy followed by biopsy. Patients who were unable to answer the questionnaire, had no histopathological report available, and presented with any type of neoplasm except non‐melanoma skin cancer were excluded from the study.

The sample size was defined based on the prevalence of *H. pylori* (69.26%) [11]. A total of 156 patients were included in the study, with a statistical power of 80% and a significance level of 0.05. The sample size calculation was performed using Stata 16.0 software.

### 2.2 Ethical Considerations

The research was approved by the Research Ethics Committee of Fundação Antônio Prudente (A.C. Camargo Cancer Center), with the consubstantiated opinion number 3.174.666 (CAAE: 53166915.9.1001.5432). All participants who agreed to participate in the study signed the informed consent form.

### 2.3 Data Collection

The patients included in this study were those who underwent upper digestive endoscopy in a reference hospital at UFG, Goiás. Data were collected using an epidemiological questionnaire. Interviews were conducted in person and remotely by previously trained researchers. The questionnaire was composed of questions related to sociodemographic data (age, sex, ethnicity, and education level), lifestyle (alcohol and tobacco consumption), and medical history (diabetes, heart attack, stroke, hypertension, anemia, chronic kidney problems, tuberculosis, and other diseases). The collected data were stored in the REDCap platform.

Age was categorized into 50 years or younger and 51–75 years. Marital status was categorized into married and unmarried (single, widowed, and divorced/separated). Education was categorized into less than 5 years (illiterate and less than 5 years), 6–12 years (6–8 and 9–12 years), high school, and higher education (high school, undergraduate, and graduate) [12]. Ethnicity was divided into two categories: White and non‐White (Brown and Black/Black) [13].

Body mass index (BMI) was categorized according to the WHO guidelines. The adult population was classified as underweight and eutrophic (≤ 18.5–24.9 kg/m^2^), overweight (25.0–29.9 kg/m^2^), and obese (≥ 30.0 kg/m^2^) [14], while the elderly population (≥ 60 years) was classified as underweight (≤ 23–28 kg/m^2^), overweight (28–30 kg/m^2^), and obese (≥ 30 kg/m^2^) [15].

The diagnosis of *H. pylori* bacteria was made by the histopathological examination of the biopsy of the gastric body and antrum. Histopathological diagnosis, which is considered the gold standard for detecting *H. pylori* , was performed at the Department of Digestive Tract of HC/UFG [16].

### 2.4 Classification of Comorbidities

Comorbidities were evaluated based on the Charlson comorbidity index (CCI), which ranks clinical conditions according to their respective scores. The CCI is a severity classification system that uses secondary diagnosis registry data to assign morbidity weights. The index assigns weights according to the existing comorbidities to obtain a total score. The final calculation was obtained by the simple sum of the points, considering that 0 = no comorbidity and ≥ 1 = presence of comorbidities [17, 18, 19]. The information on comorbidities was self‐reported by the study participants.

### 2.5 Database and Statistical Analysis

Analyses were performed using absolute and relative frequencies and measures of central tendency and dispersion. Chi‐square test and Student's *t*‐test were used to evaluate the *H. pylori* status, socioeconomic variables, and comorbidities.

Univariate and multiple logistic regression models were used for the analysis of OR and their respective 95% confidence intervals (95% CIs). The stepwise forward methodology was performed to better fit the model. For the multiple binary logistic regression models, the significant variables with a *p* value of < 0.20 and adjustment variables were used. The significance level of 5% (*p* < 0.05) was assigned for the statistical analysis. The Hosmer–Lemeshow test was performed for model fit quality analysis. Data were analyzed anonymously using the Stata 16.0 and Statistical Package for the Social Science version 25.0 software.

## 3 Results

**FIGURE 1:**
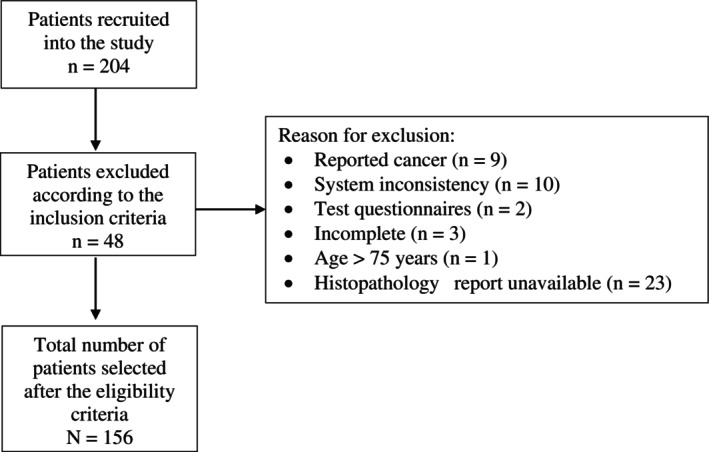
Flowchart of eligibility of the subjects in this study conducted from 2019 to 2022 in Goiás, Brazil.

In this study, of the 204 individuals, 156 participants were eligible, while 48 were excluded for not meeting the inclusion criteria (Figure 1).

The prevalence of *H. pylori* was 45.5% (71/156), wherein 60.6% (43/71) of the infected patients were female. Among the infected, 50.7% (36/71) were in the age group between 51 and 75 years; 59.2% (42/71) were married; and 40% (29/71) had education from 6 to 12 years. The most reported ethnicity was non‐White (Black/Black and Brown), with a prevalence of 70.4% (50/71) and an overweight BMI of 38% (27/71) (Table 1).

**TABLE 1:** Sociodemographic characteristics of the study population in the period from 2019 to 2022 in Goiás, Brazil.

Features	*Helicobacter pylori* negative		*H. pylori* positive		*p*
	*n*	%	*n*	%	
	85	54.50	71	45.50	
Sex					
Female	65	76.5	43	60.6	0.032^a^
Male	20	23.5	28	39.4	
Age (min–max)	18–75		18–75		0.212^b^
Age (average)	47.09 ± 15.68		50.15 ± 14.63		
Age (years)					
≤ 50	49	57.7	35	49.3	0.297^a^
51–75	36	42.3	36	50.7	
Marital status					
Married	47	55.3	42	59.2	0.628^a^
Not married	38	44.7	29	40.8	
Level of education					
Less than 5 years	14	16.5	14	19.7	0.870^a^
6–12 years	36	42.3	29	40.9	
High school and higher education	35	41.2	28	39.4	
Ethnicity					
Non‐White	69	81.2	50	70.4	0.116^a^
White	16	18.8	21	29.6	
BMI (kg/m^2^)					
Underweight and eutrophic	39	45.9	26	36.6	0.324^a^
Overweight	28	32.9	23	32.4	
Obese	18	21.2	22	31	

*Note:* BMI (kg/m^2^): body mass index. Age: ≤ 50 years (18–50 years) and 51–75 years. Marital status: married and unmarried (single, widowed, and divorced/separated). Education: less than 5 years (illiterate and less than 5 years), 6–12 years (6–8 and 9–12 years), and high school and college (high school, undergraduate, and graduate). Ethnicity: White and non‐White (Black/Black, Brown, and Asian). BMI (WHO/PAHO): underweight and eutrophic (≤ 18.5–24.9/≤ 23–28), overweight (25–29.9/28–29.9), and obese (≥ 30).

^a^ Pearson's chi‐square test.

^b^ Student's *t*‐test.

In the *H. pylori* ‐negative group, which represented 54.5% (85/156) of the sample, 76.5% (65/85) were female, 57.7% (49/85) were 50 years old or younger, and 55.3% (47/85) were married. A total of 42.3% (36/85) of the sample had education between 6 and 12 years, 81.2% (69/85) were self‐declared non‐White, and about 41.2% (35/85) were underweight or eutrophic. Among the sociodemographic variables of the groups, only gender showed a statistical difference (*p* = 0.032). On the other hand, age, marital status, education, ethnicity, and BMI showed no differences (Table 1).

**FIGURE 2:**
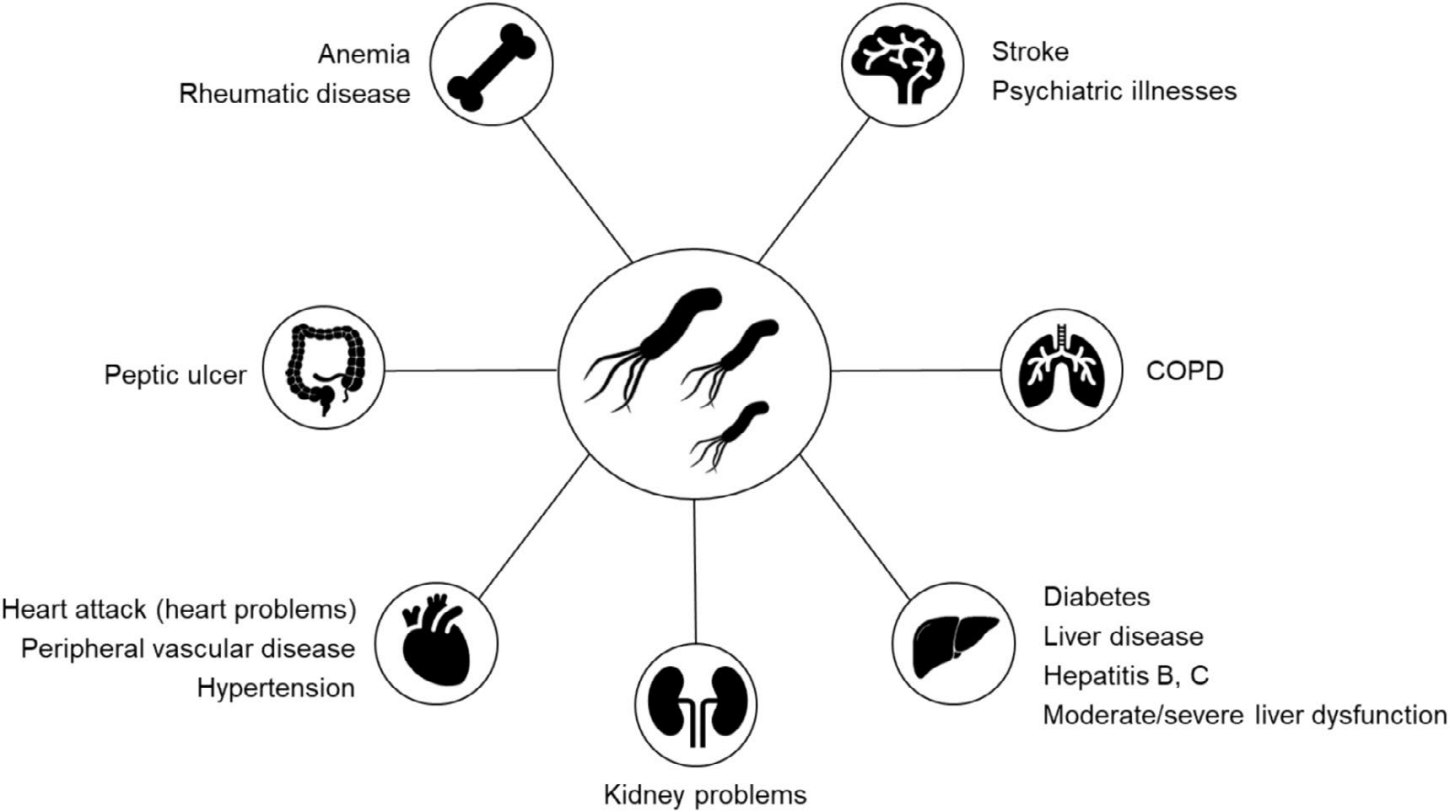
Extragastric diseases present in the population infected by *Helicobacter pylori* from 2019 to 2022 in Goiás, Brazil.

The presence of comorbidities was higher among the infected individuals (73.2%) (52/71) compared with the non‐infected individuals (68.2%) (58/85). In the *H. pylori* ‐positive group, the most common diseases were hypertension (43.7%) (31/71), anemia (29.6%) (21/71), rheumatic disease (19.7%) (14/71), and diabetes (15.5%) (11/71). In the *H. pylori* ‐negative group, the most prevalent diseases were hypertension (33%) (28/85), anemia (31.8%) (27/85), rheumatic disease (23.5%) (20/85), and psychiatric diseases (20%) (17/85) (Figure 2). It is noted that among the groups, the most commonly reported diseases were similar, differing in diabetes and psychiatric diseases (Table 2).

**TABLE 2:** Comorbidities present in the study population from 2019 to 2022 in Goiás, Brazil.

Diseases	*Helicobacter pylori* negative		*H. pylori* positive		*p*
	*n*	%	*n*	%	
Rheumatic disease					0.704^a^
Absent	65	76.5	57	80.3	
Present	20	23.5	14	19.7	
Heart attack (heart problems)					0.394^a^
Absent	73	85.9	65	91.5	
Present	12	14.1	6	8.5	
Stroke					0.456^a^
Absent	80	94.1	69	97.2	
Present	5	5.9	2	2.8	
Peripheral vascular disease					0.127^a^
Absent	79	92.9	70	98.6	
Present	6	7.1	1	1.4	
Hypertension					0.227^a^
Absent	57	67	40	56.3	
Present	28	33	31	43.7	
Diabetes					1^a^
Absent	72	84.7	60	84.5	
Present	13	15.3	11	15.5	
COPD					1^a^
Absent	74	87	62	87.3	
Present	11	13	9	12.7	
Kidney problems					0.219^a^
Absent	81	95.3	63	88.7	
Present	4	4.7	8	11.3	
Anemia					0.904^a^
Absent	58	68.2	50	70.4	
Present	27	31.8	21	29.6	
Liver disease					0.943^a^
Absent	78	91.8	64	90.1	
Present	7	8.2	7	9.9	
Moderate/severe liver dysfunction					0.729^a^
Absent	80	94.1	68	95.8	
Present	5	5.9	3	4.2	
Hepatitis B, C					0.562^a^
Absent	77	90.6	67	94.4	
Present	8	9.4	4	5.6	
Psychiatric illnesses					0.072^a^
Absent	68	80	65	91.5	
Present	17	20	6	8.5	
Peptic ulcer					0.16^a^
Absent	75	88.2	68	95.8	
Present	10	11.8	3	4.2	
CCI					0.613^a^
0	27	31.8	19	26.8	
≥ 1	58	68.2	52	73.2	

Abbreviations: CCI, Charlson comorbidity index; COPD, chronic obstructive pulmonary disease.

^a^ Pearson's chi‐square test.

Univariate analysis showed a higher chance of *H. pylori* infection in males (OR = 2.12, 95% CI: 1.06–4.22). Similarly, in the adjusted analysis, a higher chance of infection was also observed in males (OR = 2.63, 95% CI: 1.26–5.50). Ethnicity and BMI remained in the model as adjustments (Table 3).

**TABLE 3:** Univariate and adjusted logistic regression analysis of *Helicobacter pylori* infection among the study participants in the period from 2019 to 2022 in Goiás, Brazil.

Variable	Univariate OR (95% CI)	*p*	Adjusted OR (95% CI)	*p*
Sex				
Female	1	0.034	1	0.01
Male	2.12 (1.06–4.22)		2.63 (1.26–5.50)	
Ethnicity				
Non‐White	1	0.118	1	0.108
White	1.81 (0.86–3.82)		1.89 (0.87–4.10)	
BMI (kg/m^2^)				
Underweight and eutrophic	1		1	
Overweight	1.23 (0.59–2.59)	0.581	1.26 (0.58–2.71)	0.559
Obesity	1.83 (0.83–4.07)	0.136	2.31 (0.99–5.39)	0.052
Consumption of alcoholic beverages				
No	1	0.088	—	—
Yes	1.74 (0.92–3.29)		—	

*Note:* Adjusted for sex, ethnicity, and BMI. Hosmer–Lemeshow test = 0.241.

The univariate analysis observed a lower chance of psychiatric illness in *H. pylori* ‐infected individuals (OR = 0.37, 95% CI: 0.14–0.99) and a higher chance of psychiatric illness in unmarried participants (OR = 2.92, 95% CI: 1.16–7.37) with obesity (OR = 5.08, 95% CI: 1.47–17.55). In the multiple analysis, we observed a lower chance of psychiatric illness in *H. pylori* ‐positive patients (OR = 0.32, 95% CI: 0.11–0.92) and a higher chance of psychiatric illness in unmarried (OR = 2.92, 95% CI: 1.08–7.89), overweight (OR = 4.21, 95% CI: 1.16–15.30), and obese (OR = 5.70, 95% CI: 1.57–20.75) individuals. Sex, age, and alcohol consumption remained in the model as adjustments (Table 4).

**TABLE 4:** Univariate and adjusted logistic regression analysis of psychiatric disorders among the study participants from 2019 to 2022 in Goiás, Brazil.

Variable	Univariate OR (95% CI)	*p*	Adjusted OR (95% CI)	*p*
*Helicobacter pylori*				
Absent	1	0.049	1	0.034
Present	0.37 (0.14–0.99)		0.32 (0.11–0.92)	
Sex				
Female	1	0.314	—	—
Male	0.58 (0.20–1.67)		—	
Age (years)				
≤ 50	1	0.24	—	—
51–75	0.57 (0.23–1.45)		—	
Marital status				
Married	1	0.023	1	0.035
Not married	2.92 (1.16–7.37)		2.92 (1.08–7.89)	
BMI (kg/m^2^)				
Underweight and eutrophic	1	—	1	—
Overweight	3.27 (0.94–11.31)	0.062	4.21 (1.16–15.30)	0.029
Obesity	5.08 (1.47–17.55)	0.01	5.70 (1.57–20.75)	0.008
Consumption of alcoholic beverages				
No	1	0.193	—	—
Yes	0.54 (0.21–1.36)		—	

*Note:* Adjusted for *H. pylori* , marital status, and BMI. Hosmer–Lemeshow test = 0.731.

## 4 Discussion

In this study, the prevalence of *H. pylori* was 45.5%. A systematic review study with meta‐analysis showed a *H. pylori* prevalence of 63.4% in Latin America and the Caribbean and 79.1% in some countries in Africa [20]. In developing countries, such as Brazil, the prevalence of *H. pylori* can reach rates of up to 91%, especially in regions with low socioeconomic conditions [21]. In Goiás, a study conducted by the *H. pylori* Study Center on 113 dyspeptic patients observed a prevalence of 61.1%, which shows a reduction in infection [22]. These differences can be explained based on the use of different diagnostic methods, improvements in living conditions, characteristics of the study population, and their living and eating habits.

This study found that the male participants had a higher chance of being infected with the bacteria, which is consistent with other studies indicating that male sex is a potential risk factor for infection [23, 24]. In contrast, studies in China and Iran found no differences in the prevalence between genders [25, 26]. The difference in the infection prevalence between the sexes may be explained by the difference in lifestyle between men and women. Men are more exposed to risk factors for infection, such as alcohol and tobacco consumption, whereas hormonal factors, especially sex hormones, may justify the lower chance of females being infected by the bacteria.

In this study, a higher frequency of individuals who declared themselves as non‐White (Black and Brown) was observed in the *H. pylori* ‐positive and ‐negative groups. According to the Brazilian Institute of Geography and Statistics (IBGE), the Brazilian population is highly mixed; it has been estimated that about 55.3% of Brazilians declared themselves as Brown or Black [27]. In the Midwest, individuals who declared themselves as Black or Brown represent 62.6% of the population [28]. Furthermore, these findings confirm the racial‐ethnic reality of the Brazilian population.

Although the Black/Black or Brown population is the majority in Brazil (55.3%), this group experiences some difficulties mainly due to social inequality. The social conditions result in low socioeconomic status, low education, and poor quality of life. These conditions are risk factors that contribute to the acquisition of *H. pylori* infection and the development of gastric diseases [24, 29, 30].

The presence of comorbidities was higher in the *H. pylori* ‐positive group, but no statistically significant difference was observed. Similar results were found by Chang et al., in which patients without eradication therapy for *H. pylori* had a higher frequency of comorbidities [31]. Although the infected patients had a higher number of comorbidities, no statistical difference was observed between the groups, except for psychiatric diseases.

In this study, the patients in the *H. pylori* ‐negative group were more likely to have psychiatric illnesses. The *H. pylori* ‐negative patients underwent antibiotic therapy to eradicate the microorganism. Antibiotic use may be related to antibiomania, which is an adapted term to describe antibiotic‐induced manic symptoms. Although the frequency of this adverse event is rare in the literature, the number of patients who have had eradication treatment for *H. pylori* with no previous history of psychiatric disorder has increased. Clarithromycin is a semisynthetic macrolide used in association with other drugs to treat *H. pylori* and is most often related to psychotic episodes [32, 33].

Antibiotic‐related psychosis usually occurs after the first dose. The compounds generated after the metabolization of clarithromycin may be toxic to the central nervous system and increase the levels of cortisol and prostaglandins [32, 33, 34]. These side effects may account for the fact that dyspeptic *H. pylori* ‐negative individuals have some psychiatric episodes.

Furthermore, the relationship between the central nervous system and gastrointestinal function (gut–brain axis) is well established. Studies show that psychological factors, such as depressive disorders, may be associated with gastric diseases [35, 36]. Emotions, anxiety, and psychological stress may affect gastric physiology and influence gastric acid secretion, altering stomach motility [37], including in *H. pylori* ‐negative patients.

The influence of *H. pylori* infection and neuropsychiatric disorders has not yet been determined. In a study by Shiota et al., no association was observed between *H. pylori* infection and neurological disorders [38]. In contrast, different studies have demonstrated the association between *H. pylori* infection and neuropsychiatric disorders, such as depression, anxiety, Alzheimer's disease, and Parkinson's disease, among others. The infection can lead to the release of neurotransmitters, such as acetylcholine, adrenaline, and dopamine. Additionally, *H. pylori* may be associated with neuronal damage and the impaired absorption of vitamin B12 [39, 40, 41]. Although recent studies have considered *H. pylori* infection as a risk factor for psychiatric disorders, further research is needed to determine the mechanisms linking infection with psychiatric disorders.

Hypertension (AH) is a multifactorial disease caused by the interaction of environmental and genetic factors. In the present study, no association was observed between *H. pylori* infection and AH, but the infected group showed a higher prevalence of hypertension. A study conducted by Kopacova et al. on 1818 individuals also found no association between *H. pylori* infection and hypertension [42]. In contrast, Xiong et al.'s study on 17 100 participants and Huang et al.'s meta‐analysis consisting of 17 studies confirmed this association and suggested that in addition to chronic inflammation, *H. pylori* infection may cause an imbalance in lipid metabolism, generating an increase in low‐density lipoprotein levels and a reduction in high‐density lipoprotein levels [43, 44]. The evidence of this relationship has some limitations, because it is necessary to consider several factors, such as weight, age, diet, and presence of other diseases [42].

The presence of anemia was similar between the groups. This finding is consistent with the study of Shih et al. that surveyed chronic infection status and anemia in 882 adult subjects and found no significant association [45]. *H. pylori* eradication is proposed as a treatment for some cases of anemia. However, Tseng et al. reported that the eradication of the infection had no effect on individuals with unexplained iron‐deficiency anemia [46].

Although no positive association was found between *H. pylori* infection and anemia, it is described in the literature that infected individuals are 1.77 times more likely to be anemic compared with their uninfected counterparts. The possible mechanisms that may explain this association are gastrointestinal bleeding, iron sequestration by the bacteria, and reduced gastric acid secretion that is essential for dietary iron absorption [17, 47]. In Brazil, the IV Brazilian Consensus on *H. pylori* infection indicates the treatment of the infection in some cases of iron deficiency anemia, immune thrombocytopenic purpura, and vitamin B12 deficiency [48]. The positive association is usually reported in specific groups that are commonly affected by anemia, such as children, pregnant women, and the elderly.

The association between diabetes and *H. pylori* infection among adult dyspeptic patients has been investigated by different researchers [49, 50, 51]. In the present study, no statistically significant association was observed between diabetes and *H. pylori* in adult dyspeptic patients. Nevertheless, numerous authors have described that it is biologically possible for it to occur. Infection can alter the secretion of some hormones, such as ghrelin, leptin, somatostatin, and gastrin, which can interfere with the individual's insulin levels, increasing the chance of diabetes [52]. Moreover, inflammatory cytokines from the inflammation caused by *H. pylori* infection can generate insulin resistance [53, 54]. The association between *H. pylori* infection and diabetes is not well established, and other risk factors, such as genetic conditions and dietary habits, should be taken into consideration in the development of this comorbidity.

Metabolic syndrome (MetS) is a set of metabolic conditions that includes insulin resistance, hyperglycemia, dyslipidemia, obesity, and hypertension. This syndrome increases the risk of developing cerebrovascular diseases, which is an important factor for public health [55]. MetS is a consequence of chronic inflammation and altered microbiota, which may be caused by *H. pylori* infection. In fact, hypertension and diabetes were reported more frequently in the infected group, and MetS may be associated with *H. pylori* infection and these comorbidities [56, 57].

The association between bacterial infection and extragastric diseases remains controversial. Some studies suggest that the chronic inflammatory state and immunometabolic alterations induced by *H. pylori* infection may favor the emergence or worsening of metabolic and cardiovascular diseases [58, 59]. On the other hand, factors such as immune dysfunctions and pre‐existing conditions may make the host more vulnerable to infection. In fact, there is no consensus in studies as to whether infection predisposes the development of these conditions or whether individuals with comorbidities are more susceptible to bacterial colonization.

Due to the increasing evidence on the relationship between *H. pylori* infection and several comorbidities, the importance of investigating and treating this infection becomes evident. In addition to the well‐documented benefits in gastric diseases, the eradication of this bacterium may contribute to the improvement of physiological parameters associated with the aforementioned conditions. However, due to the complexity of the therapeutic regimen required for its elimination, often involving multiple drugs and long treatment periods, it is essential to assess the impact of this approach on the clinical progression of these comorbidities [60, 61].

Despite the limitations of this study, the results of our study should be interpreted under the concession of the following limitations: the sample size, the recruitment of participants in only one healthcare facility, and the absence of information about the treatment of infection. Another important limiting factor of the study is the reverse causality inherent in cross‐sectional studies. The strengths of this study are the use of histopathological diagnosis, which is recognized as the gold standard for detecting *H. pylori* , and the standardization of the interviews in the remote format due to the pandemic caused by the SARS‐CoV‐2 virus [62], which is a novelty in the scientific environment. Another point to be highlighted is that this is the first study that investigated the association between *H. pylori* infection and extragastric comorbidities in dyspeptic patients in the Midwest region of Brazil.

## 5 Conclusion

The prevalence of *H. pylori* infection was 45.5%, and males were more likely to be infected by the bacteria. The most frequent comorbidities in the *H. pylori* ‐positive group were hypertension, anemia, rheumatic disease, and diabetes. *H. pylori* ‐negative patients were more likely to have psychiatric illnesses. Thus, further research is needed to confirm the relationship between *H. pylori* infection and extragastric diseases.

## Conflicts of Interest

The authors declare no conflicts of interest.
